# Comparative analysis of plant morphometric traits, essential oil yield, and quality of *Origanum majorana* L. cultivated under diverse sustainable organic nutrient management strategies

**DOI:** 10.1038/s41598-025-20751-x

**Published:** 2025-09-25

**Authors:** Muziri Mugwanya, Fahad Kimera, Mahmoud A. O. Dawood, Osama S. Ali, Aly Reda, Tamer Shoeib, Hani Sewilam

**Affiliations:** 1 Center for Applied Research on the Environment and Sustainability (CARES), School of Science and Engineering, The American University in Cairo, AUC Avenue, P.O. Box 74, New Cairo, 11835 Egypt; 2 Animal Production Department, Faculty of Agriculture, Kafrelsheikh University, Kafr El-Sheikh, Egypt; 3 Department of Biology, School of Sciences and Engineering, The American University in Cairo, AUC Avenue, P.O. Box 74, New Cairo, 11835 Egypt; 4 Department of Chemistry, School of Sciences and Engineering, The American University in Cairo, AUC Avenue, P.O. Box 74, New Cairo, 11835 Egypt; 5 UNESCO Chair in Hydrological Changes and Water Resources Management, RWTH Aachen University, Aachen, Germany

**Keywords:** Integrated aquaculture-agriculture systems, Chemical fertilizers, Organic fertilizers, Plant nutrition, Sustainability, Plant development, Plant symbiosis, Secondary metabolism

## Abstract

Plant nutrition is one of the major factors affecting the production of medicinal plants. This study aimed to assess and compare plant morphometric characteristics, essential oil yield, and composition of *Origanum majorana* L. irrigated with water of different qualities at two cuts. The greenhouse experiment followed a randomized completely block design of three treatments (T1: water mixed with standard hydroponics solutions A and B, T2: Biofloc wastewater, and T3: aquaculture wastewater) with three replicates per treatment. The results of this study indicated that T1 significantly improved the plant morphometric characteristics, such as plant height, number of branches, fresh weight, and dry weight, compared to other treatments in cut 1 and cut 2. The results of the soil analysis showed that T1 had higher values for the soil bulk density, potassium content, and electroconductivity compared to other treatments. The total phenolic content was significantly higher in T2 than in T1 and T3 in both cuts. However, the essential oil yield in cut 2 was higher than that in cut 1 across all the treatments, with T1 recording higher values, followed by T2 and T3, respectively. The major essential oil constituents in all the treatments were 4-Terpineol (33.95% − 50.24%), γ-Terpinene (3.95% − 15.74%), α-Humulene (3.39% − 10.33%), α-Terpineol (3.72% − 8.37%), and Sabinene (2.16% − 6.31%). In cut 1, the percentage of the major essential oil constituents was in the order T1 > T2 > T3, versus cut 2, which was T3 > T1 > T2. In conclusion, irrigating *Origanum majorana* with hydroponic solutions enhances plant growth, essential oil yield, and composition. However, irrigating *Origanum majorana* with Biofloc wastewater could be a feasible alternative for the organic production of marjoram and other high-value medicinal herbs in a sustainable approach.

**Supplementary Information:**

The online version contains supplementary material available at 10.1038/s41598-025-20751-x.

## Introduction

Recently, the list of plants of economic significance has been extended to include species with desired aromatic and medicinal properties^1–3^. Since the beginning of time, people have always used medicinal and aromatic plants (MAPs) in folk medicine due to their therapeutic properties in tackling different ailments and diseases with negligible side effects^4–6^. MAPs contain several bioactive compounds such as phenolics, flavonoids, and essential oils (EOs) in different concentrations, thus crucial active ingredients that have shaped modern medicine^7–9^. However, according to the World Health Organization (WHO), 65–80% of people living in rural areas and low-income countries still rely on MAPs in the treatment of several diseases and injuries, often due to poverty and lack of access to modern medicine^10,11^.

Besides their medicinal potential, MAPs are also desired for their aroma and flavor, thus significant contributors to the global chemical trade^12,13^. The current substantial growth in the global demand for MAPs has offered exporting countries a chance to improve their industrial production through different agronomical practices and processing techniques to meet the global demand^12^. According to FAOSTAT^14^, the global cultivated and harvested areas of MAPs stood at 12.7 million hectares and 90.8 million tons, respectively. Moreover, the European Union (EU) imports MAPs from Africa and Asia worth ~ 1 billion USD^15^. In the same regard, recent data indicates that the global MAPs market is expected to grow from $215.4 billion in 2024 to $375.6 billion by 2032^16^. In the Middle East and North Africa, Egypt is one of the major producers and exporters of MAPs, with available data indicating that the harvested area and yield reached up to ~ 32,507 hectares and 0.888 tons/hectare, respectively, as of 2021^17^. Among the most widely cultivated and exported MAPs is sweet marjoram (*Origanum majorana*), whose cultivation in Egypt dates back to 3000 BC. The Egyptian marjoram has a good global market reputation due to the high quality of its EOs, and in 2020, Egypt was ranked 9th in the international exportation of marjoram with an export value of 100.57 million^18^. The Egyptian government, therefore, aims to increase the area of MAPs in newly reclaimed lands to increase production.

Newly reclaimed lands in Egypt contain sandy soil that is poor in nutrient elements, low in organic matter, and has a low water-holding capacity, thus low crop yield^19,20^. Proper application of organic fertilizers enhances the soil structure, nutrient composition, and crop yield^21–24^. Moreover, wastewater from aquaculture and other fish production systems, such as Biofloc Technology (BFT), is rich in organic matter and nutrient elements such as nitrogen (N), phosphorus (P), and potassium (K), which are important for plant growth and development^25–28^. BFT systems refer to self-sustaining ecosystems of microbes held together in a complex matrix called Bioflocs, which act as a natural wastewater purification system within the aquaculture pond or tank^27^. Previous studies have shown that irrigating crops with aquaculture wastewater enhances plant growth and yield as well as the physiochemical properties of the soil^26,29,30^. Likewise, the application of aquaculture sludge as a biofertilizer has been shown to improve the organic matter content of the soil as well as improve the yield of several crops^31–33^. Similarly, the integration of BFT with hydroponics and or/irrigating plants with BFT wastewater has been shown to improve the growth and yield of several plants^28,34–36^.. To the best of our knowledge, no comparative study has been made on the influence of irrigating *O. majorana* with aquaculture and BFT wastewater under greenhouse conditions. The aim of this study, therefore, is to assess the variation in morphometric characteristics, EO yield and composition of *O. majorana* irrigated with hydroponics solutions A and B, aquaculture wastewater, and BFT wastewater.

## Materials and methods

### Experimental design

**Fig. 1:**
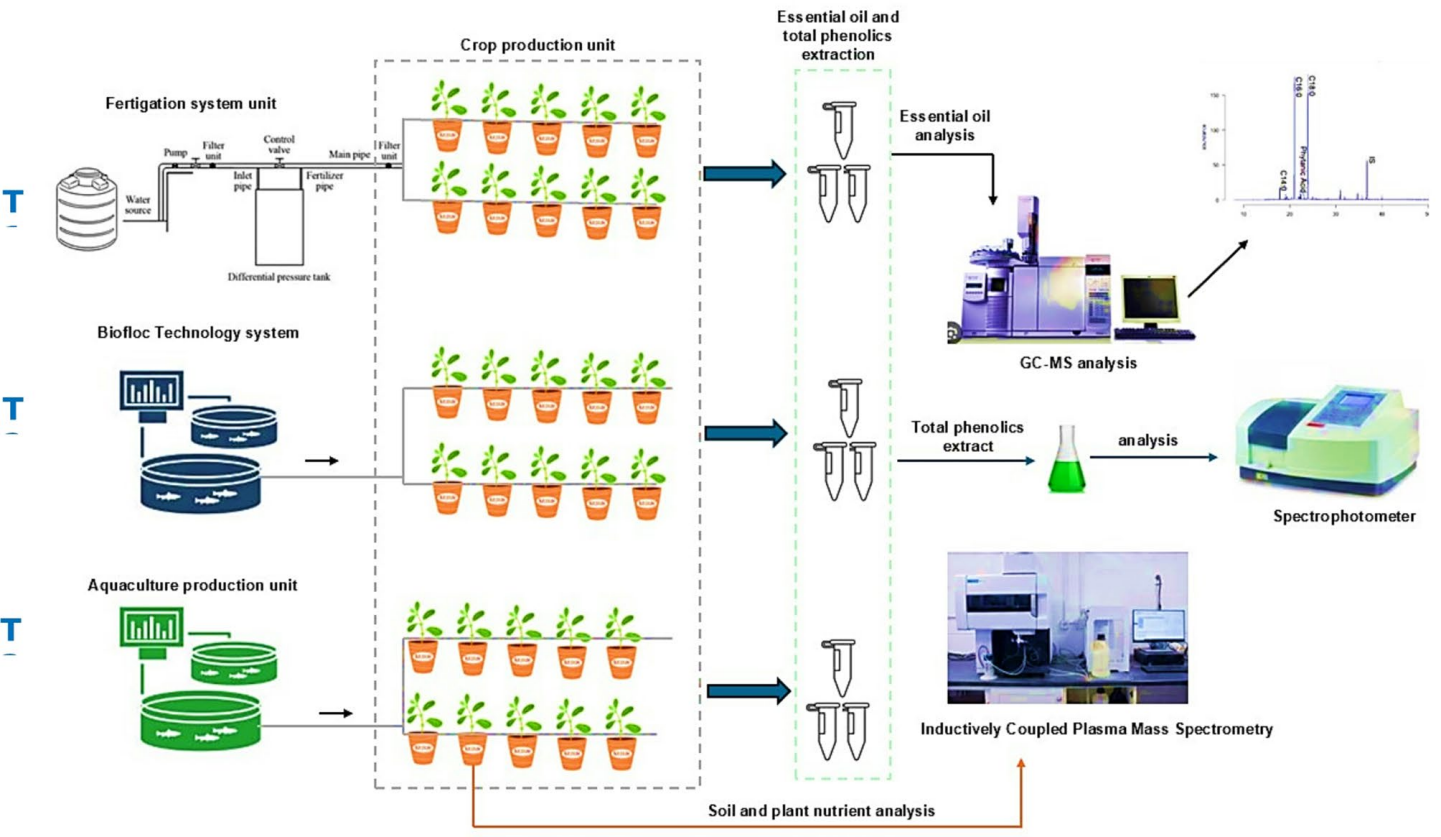
Schematic Experimental set-up and treatments.

Seedlings of *Origanum majorana* L. were purchased from a local distributor and botanically identified at the Plant Classification Department of the Agricultural Research Center in Giza, Egypt. They were then transplanted into sterilized greenhouse plastic pots (30 cm in diameter) filled with sand. The pots were connected to 1000 L tanks by drip irrigation pipes, supplying water of different qualities and the experiment was laid out in a randomized completely block design of three treatments (T1, T2, and T3) with three replicates. The treatments were as follows. T1: water mixed with hydroponics solutions (solutions A and B), T2: Biofloc wastewater, and T3: aquaculture wastewater (Fig. 1). The greenhouse layout of our experimental treatments is presented in **Supplementary Fig. 1**. In T2 and T3, Nile tilapia (*Oreochromis niloticus*, initial weight 54.29 g) was stocked at a stocking density of 70 fish/m^3^. Fish were fed to satiation on a commercial diet containing 30% protein. To activate the formation of flocs, 88.92 g of molasses was added in T2 under constant aeration, and an Imhoff cone was used to monitor the volume and maturation of flocs, which were kept between 20 and 40 ml/L by either stopping the addition of molasses or replacement of 1% of the total volume of water channeled to the pots via drip irrigation pipes. The pipes were periodically maintained to avoid blockage. Plants were irrigated once per day for 3 minutes with a dripper flow rate of 4 L per minute.

### Wastewater quality measurement

Wastewater quality parameters such as water temperature, total dissolved solids (TDS), and pH were monitored daily and measured using hand-held devices. For quantification of ammonia, nitrite, and nitrate concentrations, water samples were collected twice a week in the early morning before feeding and immediately taken to the lab for analysis using HANNA reagent kits for ammonia (H193715-01), nitrite (H193707-01), and nitrates (H193728-01) as previously described by Kimera et al.^37^.

### Plant morphometric characteristics

Six plants of uniform growth per replicate per treatment were randomly tagged for data collection. Plant heights were measured from the crown of the plant to the upper part of the plant using a meter rule. The number of branches was counted, and averages were determined. Aerial plant parts (shoots and leaves) were obtained and weighed, and averages were recorded as fresh weight in g/plant. Samples were then dried at 60 °C for 72 h to obtain dry weights.

### Extraction, quantification, and analysis of essential oils

The essential oils (EOs) were extracted via steam distillation with a Clevenger apparatus; 300 g of fresh aerial parts of the plant were heated in the Clevenger flask for 3 h. The extracted EOs were then dehydrated using sodium sulfate and weighed precisely. The percentage of the extracted EOs was determined based on the weight of the oil extracted from 100 g of plant material (w/w). The EOs were kept in dark glass vials at 4 °C until further analysis. For the EOs composition analysis, an Agilent gas chromatograph (Model Agilent 5977B GC/MSD) from Agilent Technologies (Santa Clara, CA, USA), coupled with a single Quadrupole-MS mass spectrometer, was used. EOs compounds were separated using an MP-5MS column (Agilent). The ionization energy was set at 70 EV, and the ionization source temperature was maintained at 280 °C. The set point was held at 40 °C for 1 min, followed by an increase to 150 °C at a rate of 4 °C/min, then maintained for 6 min. Subsequently, the temperature further increases to 210 °C at a rate of 4 °C/min, then held for 1 min.

Helium was used as the carrier gas, flowing at 1 mL/min. The injector and interface temperatures were maintained at 280 and 220 °C, respectively, with a split ratio of 1:10. The identification of the essential oil constituents was achieved by determining their retention indices through temperature-programmed analysis with n-alkanes (C_8_–C_20_) and comparing retention indices with literature values by comparing retention times to authentic standards and/or (3) matching mass spectra against internal libraries (NIST17)^38^.

### Extraction and quantification of total phenolics

The extraction and quantification of total phenolics was performed according to the methods described by Yarahmadi et al.^38^. Briefly, 10 g of dried and ground plant material were mixed with 100 mL of an 80% methanol solution. The mixture was then shaken at room temperature for 72 h, followed by filtration through filter paper. Methanol was removed from the collected plant extract using a rotary evaporator (Buchi). The resulting pure extract was then stored in glass vials at 4 °C until further analysis for total phenolic content (TPC). The TPC was determined according to AOAC 2017.13 with some modifications by mixing the plant extracts with 400 µL of 10% Folin–Ciocalteu reagent and 2 mL of 10% sodium carbonate (Na_2_CO_3_) solution. The mixtures were incubated at 40 °C for 15 min. Absorbance was recorded at 765 nm using an Agilent Cary 3500 double-beam spectrophotometer. All measurements were taken in duplicates. The total phenol content was calculated as mg gallic acid equivalent (GAE) per gram of dry weight (DW) using a standard curve of gallic.

### Quantification of macroelements

Macroelements (i.e. phosphorus (P) and potassium (K)) were determined as described by Nguyen et al.^39^. with slight modifications. Briefly, 300 mg samples were obtained for microwave digestion (Model: Speed Wave Entry DAP60 K). Digested samples were placed in a digestion vessel, and 65% 3.0 ml of nitric acid (HNO_3_) and 7.0 ml of 35% hydrochloric acid (HCl) were added. The mixture was carefully stirred, and samples were heated in the microwave following a standard program. After microwave digestion, the samples were cooled, and the resultant clear solution was used for the analysis of P and K using an inductively coupled plasma–optical emission spectroscopy (ICP-OES, Model: Thermo ICAP 7400). For quantification of nitrogen (N) content, samples were shade-dried in a ventilated area to retain their nitrogen levels. Once dried, the samples were ground and sieved to ensure a consistent particle size. 0.2 g of each sample was placed in tin foil capsules and analyzed in duplicate. The total nitrogen content was determined using the Dumas direct combustion method with a LECO FP 528 protein/nitrogen determination^40^. The total protein content was obtained by taking the determined nitrogen amount and multiplying it by the suitable protein conversion factor^41^.

### Soil analysis

At the end of the experimental period, three soil samples per replicate per treatment were pooled and taken to the Soil and Water Analysis Research Institute, Giza, Egypt, for the pH, electroconductivity (EC), N, P, and K analysis as previously described by Sewilam et al.^42^. Soil texture and structure were determined as described by Beretta et al.^43^.

### Statistical analysis

Statistical analysis was conducted using R Statistical Programming Language (version 4.4.1). All datasets on plant morphometric characteristics were checked for normality and equality of variances using the Shapiro-Wilk and Levene’s tests, respectively. Analysis of the Variance (ANOVA) test was conducted to test for significant differences between the treatments in each cut at *p* < 0.05. The Duncan Multiple Range Test (DMRT) was used to compare differences among the treatment means at *p* < 0.05. T-tests were conducted to compare treatment means between the two cuts. Datasets were normalized for the computation of the Principal Component Analysis (PCA) and construction of heatmaps. Results were visualized in MS Excel and R using R packages (ggplot2 and corrplot).

### Plant material

All plant materials and related procedures in this study were done in accordance with the guidelines of the Institutional Review Board of the American University in Cairo and the Ministry of Agriculture and Land Reclamation in Egypt.

### Ethics approval

This study followed the guidelines and approval of the Committee of Animal Welfare and Research Ethics, Faculty of Agriculture, Kafrelsheikh University, Egypt. We confirm that all methods were performed in accordance with the relevant guidelines and regulations, including the ARRIVE guidelines (https://arriveguidelines.org/) to ensure the ethical treatment and welfare of the animals used in this study.

## Results

### Wastewater quality

**Fig. 2:**
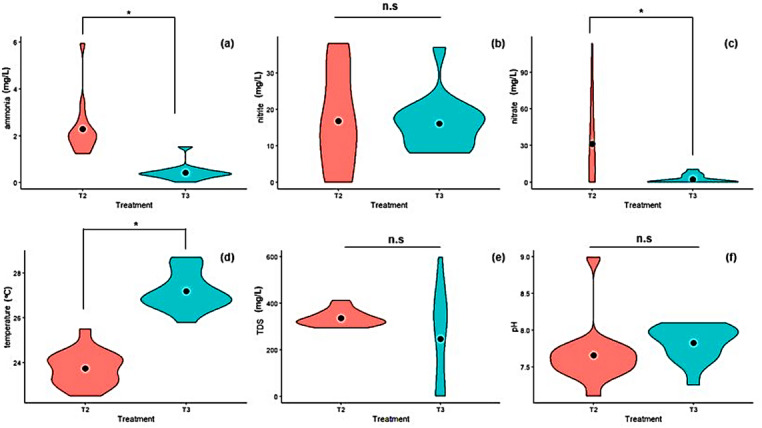
Violin plots of wastewater quality parameters measured throughout the experimental period. Black dots indicate mean values. Asterisks indicate significant differences at *p* < 0.05. T2: Biofloc wastewater treatment and T3: aquaculture wastewater treatment.

The wastewater quality from T2 and T3 was measured throughout the experimental period. Results of the measured parameters are presented in Fig. 2. The ammonia concentration ranged from 0.43 mg/L to 2.28 mg/L with T2 significantly (*p* < 0.05) recording higher values than T3 (Fig. 2a). Similarly, T2 significantly (*p* < 0.05) recorded higher for the nitrate concentration than T3 (Fig. 2c). However, the water temperature in T3 was significantly (*p* < 0.05) higher than that recorded in T2 (Fig. 2d). No significant differences were observed in the concentration of nitrites (Fig. 2b), total dissolved solids (TDS) (Fig. 2e), and pH (Fig. 2f).

### Plant morphometric characteristics

**Fig. 3:**
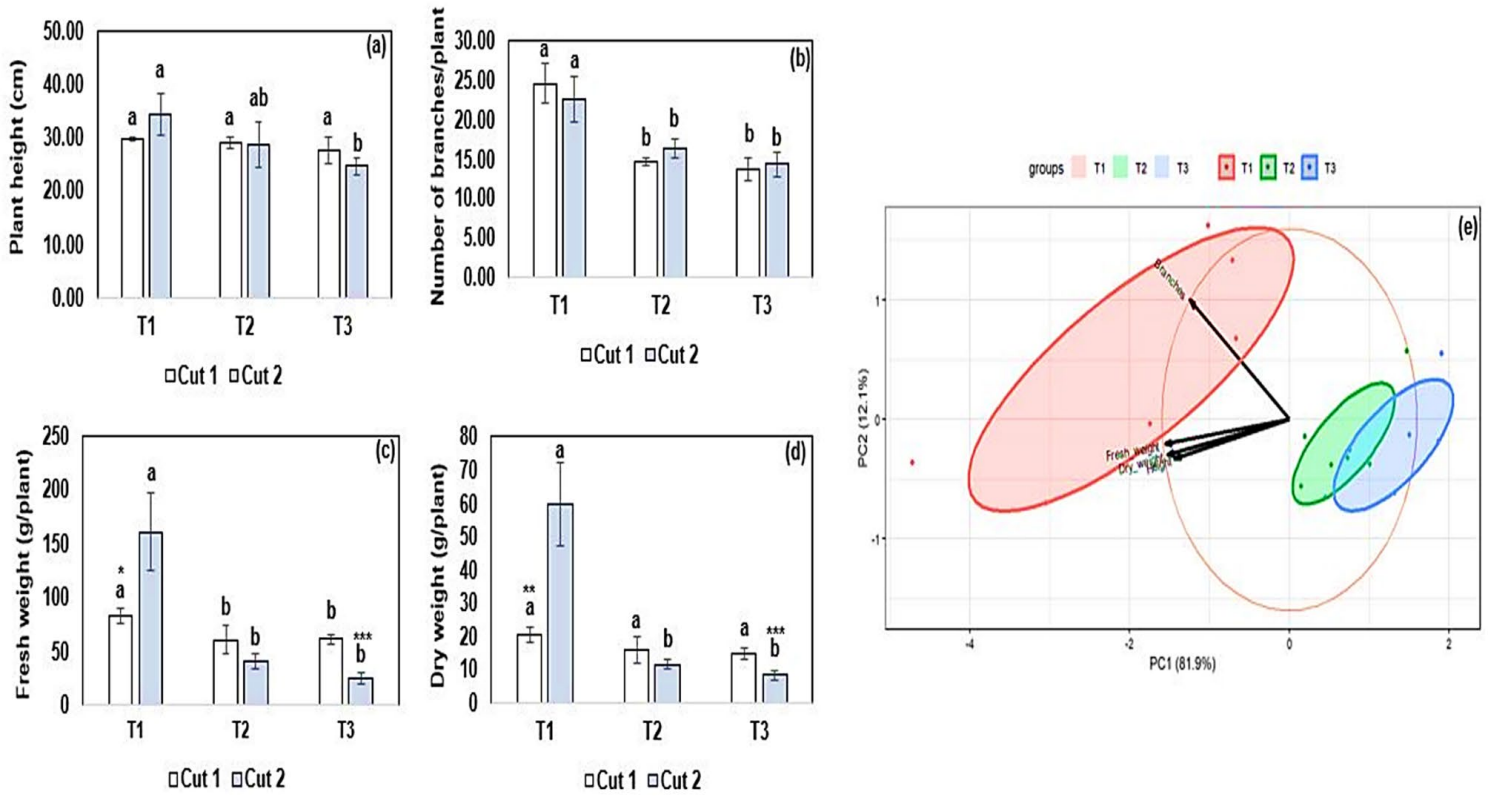
Biometric characteristics of *Origanum majorana* cultivated under different treatments (a – d). Data is presented as mean ± standard error (SE). Error bars indicate the SE. Bar columns having different letters are significantly different (*p* < 0.05). Asterisks indicate significant differences within treatments in cut 1 and cut 2 at * *p* < 0.05, ** *p* < 0.01, ** *p* < 0.001. Principal component analysis biplot (e) indicates the similarities and differences in the biometric characteristics of *O. majorana*. T1: freshwater mixed with hydroponics solutions A and B treatment, T2: Biofloc wastewater treatment, and T3: aquaculture wastewater treatment.

The results of the plant morphometric characteristics of *Origanum majorana* cultivated under different water qualities are shown in Fig. 3. For plant height (Fig. 3a), no significant difference was noted among the treatments in cut 1. However, in cut 2, T3 significantly (*p* < 0.05) recorded lower values for plant height compared to T1. Overall, no significant differences in plant heights were noted in cut 1 and cut 2 within each treatment. For the number of side branches (Fig. 3b), T1 significantly (*p* < 0.05) recorded the highest value compared to other treatments in both cuts. Overall, no significant differences in the number of side branches were noted in cuts 1 and 2 within each treatment. Data on fresh weights (Fig. 3c) indicated that T1 significantly (*p* < 0.05) had the highest value compared to other treatments in both cuts. By comparing fresh weights in cut 2 and cut 1, the results indicate that T1 and T3 significantly (*p* < 0.05) recorded higher and lower values, respectively, in cut 2 than in cut 1. For the dry weights (Fig. 3d), T1 significantly (*p* < 0.05) recorded the highest values in cut 2 compared to other treatments. By comparing dry weights in cut 2 and cut 1, the results indicate that T1 and T3 significantly (*p* < 0.05) recorded higher and lower values, respectively, in cut 2 than in cut 1.

The variations in the plant morphometric characteristics were investigated by a principal component analysis (PCA) as shown in Fig. 3e. PC1 and PC2 represented 81.9% and 12.1% of the total variance, respectively. As shown by the PCA biplots, there was an overlap of the biplots of T2 and T3, indicating shared similarities in the morphometric characteristics of *O. majorana*.

### Nutrient composition, essential oil yield, and total phenolic content

**Fig. 4:**
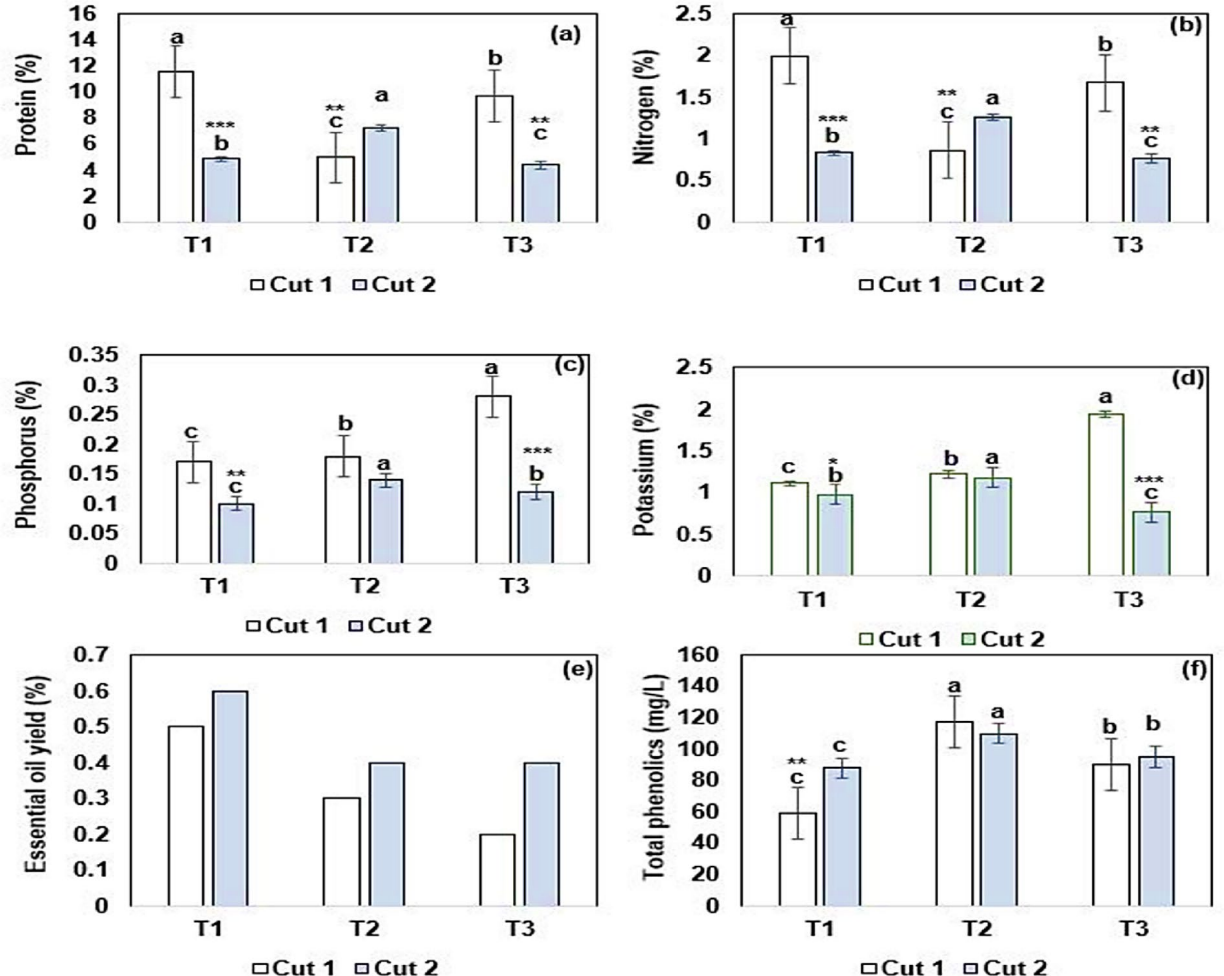
Bar graphs of nutrient composition (a, b, c, d), essential oil yield (e), and total phenolics content (f) of *Origanum majorana* cultivated under different treatments. Data is presented as mean ± standard error (SE). Error bars show the SE. Bar columns having different letters are significantly different (*p* < 0.05). Asterisks indicate significant differences within treatments in cut 1 and cut 2 at * *p* < 0.05, ** *p* < 0.01, ** *p* < 0.001. T1: freshwater mixed with hydroponics solutions A and B treatment, T2: Biofloc wastewater treatment, and T3: aquaculture wastewater treatment.

Figure 4 summarizes the nutrient composition, essential oil yield, and total phenolic content (TPC) of *O. majorana* cultivated under different treatments. In cut 1, T1 significantly (*p* < 0.05) recorded the highest protein (Fig. 4a) and nitrogen contents (Fig. 4b), followed by T2 and T3, respectively. However, in cut 2, T2 had significantly (*p* < 0.05) higher values for the protein and nitrogen content compared to T1 and T3. For the phosphorus (Fig. 4c) and potassium (Fig. 4d), T3 significantly (*p* < 0.05) recorded higher values compared to T2 and T1 in both cuts. Results of the essential oil (EO) yield (Fig. 4e) show that T1 had the highest EO yield compared to other treatments in both cuts. However, the total phenolic content (TPC) was significantly (*p* < 0.05) higher in T2, followed by T3 and T1 respectively (Fig. 4f). Overall, cut 2 significantly (*p* < 0.05) recorded lower values for the protein, nitrogen, and phosphorous contents than cut 1 across all treatments. However, a higher EO yield was noted in cut 2 than in cut 1 across all treatments.

### Pearson correlation

**Fig. 5:**
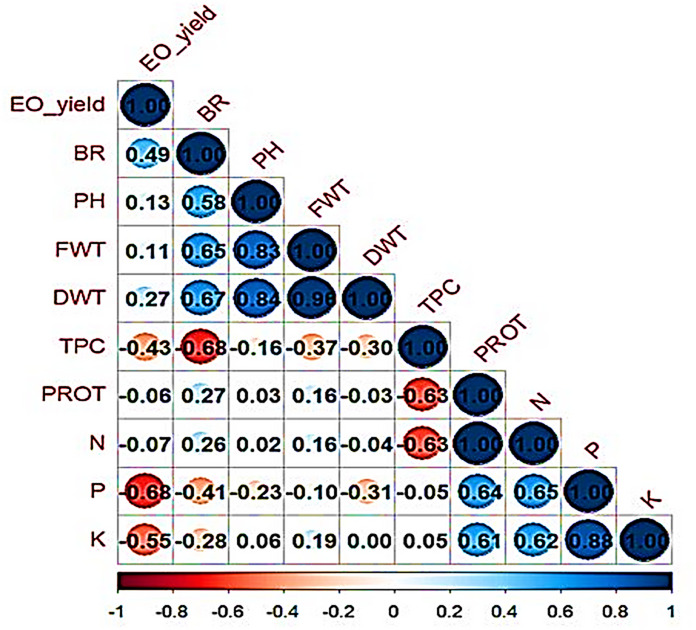
A correlogram showing correlation analysis of the biometric characteristics, nutrient content, total phenolic content, and essential oil yield of *Origanum majorana*. Blue and brown colors are positive and negative significant correlations, respectively, according to Pearson’s correlation analysis. The color intensity and circle size are proportional to the correlation coefficient. PH: plant heights, BR: side branches, FWT: fresh weight, DWT: dry weight, TPC: total phenolic content, EO_yield: essential oil yield, PROT: proteins, N: nitrogen, P: phosphorus, and K: potassium.

The positive and negative correlations of the plant morphometric characteristics, nutrient composition, EO yield and TPC are shown in Fig. 5. The potassium content showed moderate and positive correlations to the protein (*r* = 0.61) and nitrogen (*r* = 0.62) contents as well as a strong and positive correlation to the phosphorus content (*r* = 0.88). Likewise, the phosphorus content showed moderate and positive correlations to the protein (*r* = 0.64) and nitrogen (*r* = 0.65) content. The nitrogen content showed a strong and positive correlation to the protein content (*r* = 1.00). The dry weight showed a moderate and positive correlation to the number of branches (*r* = 0.67), whereas it was strongly and positively correlated to the plant height (*r* = 0.84) and fresh weight (*r* = 0.98). Similarly, the fresh weight showed a moderate and strong positive correlation to the number of branches (*r* = 0.65) and plant height (*r* = 0.83), respectively. The EO yield showed a weak and positive correlation to the number of branches (*r* = 0.49), plant height (*r* = 0.13), fresh weight (*r* = 0.11), and dry weight (*r* = 0.27). However, the TPC was negatively correlated with all other parameters.

### Essential oil composition

**Fig. 6:**
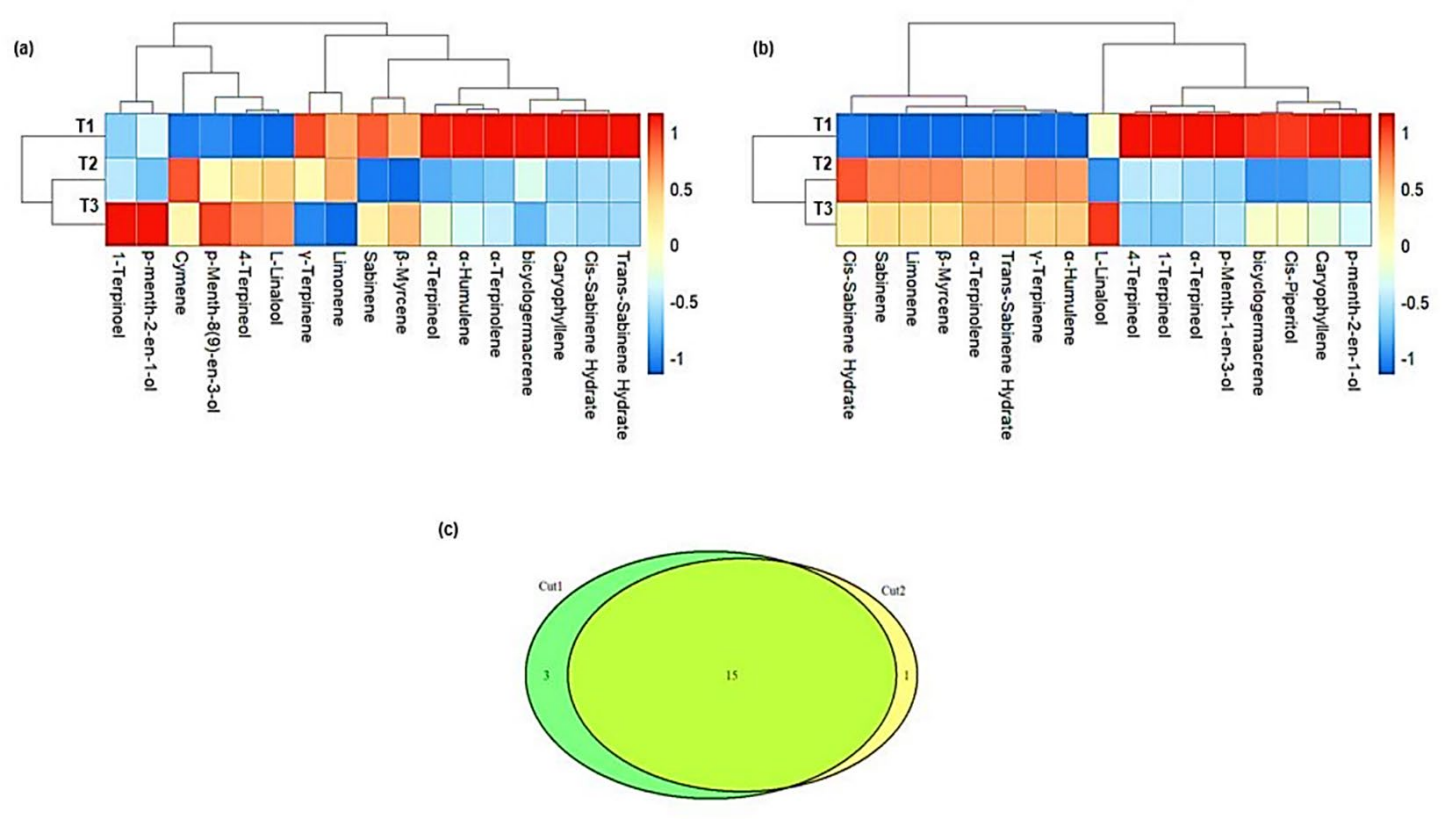
Hierarchical clustering dendrogram and heat map for essential oil constituents of *Origanum majorana* under different irrigation water quality treatments in cut 1 (a) and cut 2 (b). Colors represent a relative scale (− 1 to + 1). The darker blue indicates lower values, while the darker red indicates higher values. A Venn diagram of unique and shared essential oil constituents in cut 1 and cut 2 (c). T1: freshwater mixed with hydroponics solutions A and B treatment, T2: Biofloc wastewater treatment, and T3: aquaculture wastewater treatment.

The results of the EO composition of *O. majorana* cultivated under different treatments at two cuts are presented in Fig. 6. In the same regard, the retention index and retention area of the EO constituents are presented in Table 1. In cut 1, the dominant EO constituents of T1 were α-Terpinoel, α-Humulene, α-Terpinolene, bicyclogermacrene, caryophyllene, Cis-Sabinene Hydrate, and Trans-Sabinene Hydrate. In T2 the dominant EO constituent was cymene, whereas in T3 were 1-Terpinoel, p-menth-2-en-ol, and p-Menth-8(9)-en-3ol (Fig. 6a). For cut 2, the results of this study show that the dominant EO constituents in T1 were 4-Terpinoel, 1-Terpinoel, α-Terpinoel, p-menth-1-en-3ol, bicyclogermacrene, Cis-Piperitol, caryophyllene, and p-menth-2-en-ol. In T2, the dominant EO constituent was Cis-Sabinene-Hydrate, whereas in T3 was L-Linalool (Fig. 6b). Overall, a total of 15 EO constituents were shared between cut 1 and cut 2, whereas cut 1 and cut 2 uniquely contained 3 and 1 EO constituents, respectively (Fig. 6c).

**Table 1:** The retention index and retention area of essential oil constituents of *Origanum Majorana* cultivated under different water qualities in cut 1 and cut 2, respectively. RI: retention index, RA: retention area, T1: freshwater mixed with hydroponics solutions A and B treatment, T2: Biofloc wastewater treatment, and T3: aquaculture wastewater treatment. These essential oil constituents comprise at least 1% of the RA in any of the irrigation water quality treatments.

	T1	T2	T3	
Essential oil constituent	RA (%)	RA (%)	RA (%)	RI
**Cut 1**				
4-Terpineol	50.24	54.57	34.25	1183–1185
α-Terpineol	3.72	4.20	5.19	1193–1195
1-Terpineol	1.81	2.45	1.74	1122–1123
L-Linalool	1.39	1.52	0.54	1101–1102
p-Menth-2-en-1-ol	1.04	1.39	1.11	1141–1142
p-Menth-8(9)-en-3-ol	0.67	1.29	---	1199
Cis-Sabinene Hydrate	0.08	0.03	7.70	1098–1100
Trans-Sabinene Hydrate	0.11	0.07	3.16	1067–1068
Cymene	10.99	6.61	0.67	1024–1026
λ-Terpinene	9.38	3.95	13.87	1060–1061
Sabinene	3.51	4.82	5.62	972–974
Limonene	2.99	2.62	2.99	1028–1030
α-Terpinolene	2.60	2.70	3.34	1088–1089
β-Myrcene	1.12	1.49	1.50	991–993
α-Humulene	3.39	4.45	7.97	1016–1017
Caryophyllene	0.66	0.73	1.77	1421–1422
Bicyclogermacrene	0.48	0.80	1.63	1498–1500
**Cut 2**				
4-Terpineol	35.15	33.95	46.39	1183–1185
α-Terpineol	5.11	5.12	8.37	1193–1195
1-Terpineol	1.92	1.87	2.23	1122–1123
L-Linalool	1.23	1.43	1.32	1101–1102
p-Menth-2-en-1-ol	1.31	1.39	1.69	1141–1142
Cis-Sabinene Hydrate	3.30	1.87	0.19	1098–1100
Trans-Sabinene Hydrate	1.24	1.20	0.17	1067–1068
p-Menth-1-en-3-ol	0.78	0.80	1.11	1197–1198
Cis-Piperitol	0.76	0.93	1.14	1209
λ-Terpinene	15.74	14.97	12.77	1060–1061
Sabinene	6.31	5.48	2.16	972–974
Limonene	3.54	3.26	2.05	1028–1030
α-Terpinolene	4.04	3.99	2.93	1088–1089
β-Myrcene	1.67	1.47	0.60	991–993
α-Humulene	10.33	9.76	4.66	1016–1017
Caryophyllene	1.66	2.30	3.71	1421–1422
Bicyclogermacrene	2.31	3.45	4.98	1498–1500

RI: retention index, RA: retention area, T1: freshwater mixed with hydroponics solutions A and B treatment, T2: Biofloc wastewater treatment, and T3: aquaculture wastewater treatment. These essential oil constituents comprise at least 1% of the RA in any of the irrigation water quality treatments.

### Principal component analysis

**Fig. 7:**
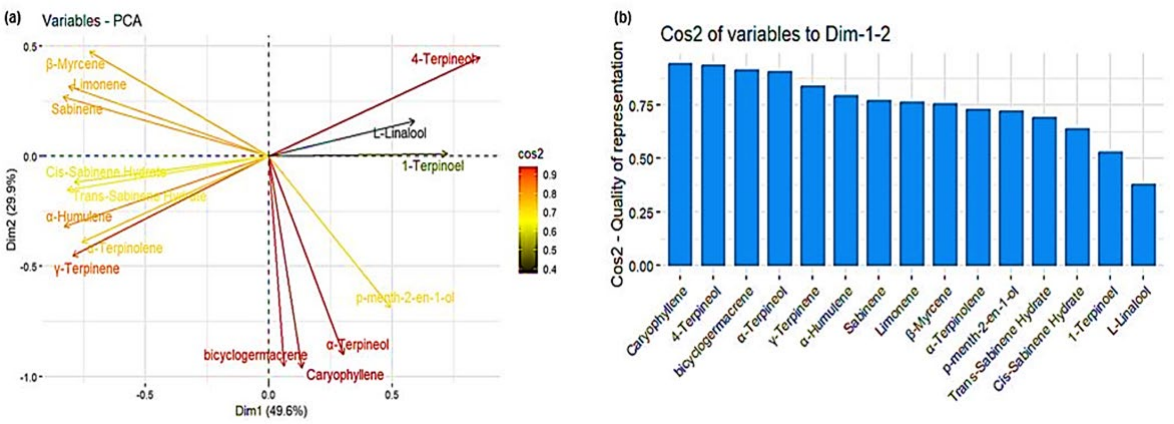
(a) The principal component analysis (PCA) according to the shared essential oil constituents of Origanum majorana and (b) Cos2 values of the essential oil constituents.

The principal component analysis (PCA) was performed to elucidate the associations of EO constituents of *O. majorana* to show that a considerable portion of observed variability (79.5%) could be explained by the first two components (Fig. 7). As indicated by the PCA results, the contribution of the first and second axes to the EO constituent’s variance was found to be 49.6% and 29.9% respectively (Fig. 7a). The results also show that EO constituents such as caryophyllene, 4-Terpineol, bicyclogermacrene, and α-Terpinoel had a good representation of the components as indicated by high cos2 values whereas 1-Terpinoel and L-Linalool had the least representation of the components as indicated by low cos2 values (Fig. 7b). Furthermore, the results show that limonene and sabinene are positively correlated are not correlated with Cis-Sabinene Hydrate, Trans-Sabinene Hydrate, α-Humulene, α-Terpinolene, and λ-Terpinene. Likewise, bicyclogermacrene, caryophyllene, and α-Terpineol are positively correlated but not correlated with 4-Terpineol, L-Linalool, and 1-Terpineol.

### Soil analysis

The soil physiochemical properties and soil classification are summarized in Table 2. The results show that the pH of all the soil samples was 7.8, and the electroconductivity ranged from 1.8 to 3.5 dS/m, with higher values recorded in T1. T3 recorded the highest and lowest values for nitrogen and phosphorus content. However, T1 recorded higher values for the potassium content compared to other treatments.

Results of the soil classification showed that T1 had a higher percentage of clay and sand compared to T3. Likewise, T1 recorded a higher value for the bulk density, followed by T2 and T3, respectively. Overall, the soil texture from all the treatments was loamy sand.

**Table 2:** Physiochemical properties and classification of soil.

Treatment	Physio-parameters		Macroelements (mg/kg)			Soil classification				
	pH	EC (dS/m)	*N*	*P*	K	Clay	Silt	Sand	Texture	B.D
T1	7.8	3.5	32.0	6.9	57.0	77.5	20.0	2.5	Loamy sand	1.6
T2	7.8	1.8	34.0	9.0	37.0	76.5	21.0	2.5	Loamy sand	1.5
T3	7.8	1.8	48.0	6.4	37.0	77.0	21.5	1.5	Loamy sand	1.4

EC; electroconductivity, N; nitrogen, P; phosphorous, K; potassium, B.D; bulk density, T1; freshwater mixed with hydroponics solutions A and B treatment, T2; Biofloc wastewater treatment, and T3; aquaculture wastewater treatment.

## Discussion

*Origanum majorana* is one of the most cultivated medicinal and aromatic plants (MAPs) around the world^44^. It is rich in several bioactive compounds of known pharmacological and medicinal properties, such as antimicrobial, antiviral, and antioxidant properties, among others^3,45,46^. The increase in bioactive compounds, essential oil yield, and quality is the major objective of MAP agriculture. Thus, several agronomical practices, such as drip irrigation with wastewater from different sources, fertigation, and multiple cuts, have been proposed to achieve these objectives.

In this study, better plant morphometric characteristics, such as plant heights, number of branches, fresh weight, and dry weight, were achieved in plants irrigated with a mixture of freshwater with hydroponic solutions A and B (T1) compared to those irrigated with Biofloc wastewater (T2) and aquaculture wastewater (T3). Moreover, higher values were recorded in cut 2 than in cut 1; hence, similar to previous studies on *Ocimum*sp^42^., *Origanum syriacum*L^47^., *Dracocephalum moldavica*, *Hyssopus officinalis*, and *Salvia officinalis*^48^. This can be attributed to the fact that after the first cut, vegetative growth initiates from an existing and well-established root system, which boosts vegetative growth^48–50^. Plants in T1 reached a maximum height of 34.33 cm, which is within the range of the maximum height (30–100 cm) attained by *Origanum*sp^50^.. However, the low values recorded for plant heights in T2 and T3 could be due to limitations in the bioavailability of certain macroelements such as nitrogen (N). According to the literature, N promotes both the multiplication and elongation of internodes, which culminates in the progressive increase in plant height^51,52^, hence any limitations in its bioavailability can negatively impact plant growth. Furthermore, regardless of treatments and cut numbers, the number of branches, fresh weights, and dry weights reported in this study were lower than those reported in previous studies^50,53,54^. The discrepancy in results is attributed to differences in experimental conditions, such as the source of fertilizers and weather conditions. For instance, biofertilizers and organic fertilizers such as manure, vermicompost, and compost have been previously reported to boost the vegetative growth of several MAPs, such as *Origanum vulgare*^55–57^, *Thymus vulgaris* L. and *Salvia officinalis*L^58^., *Plectrantus amboinicus*^59^, *Foeniculum vulgare*^60^, *Coriandrum sativum*L^61^., *Andrographis paniculata*^62^, and *Cichorium intybus*L^63^.. Weather parameters such as solar radiation (i.e. photosynthetically active radiation [400–700 nm] and ultraviolet radiation [280–315 nm]) and air temperature (20–28 °C) have a significant impact on the growth and productivity of MAPs^64,65^ hence, any conditions below the optimum threshold, especially under greenhouse conditions, negatively impact the plant morphometric characteristics.

Plants respond to environmental stress via several biochemical pathways, among which is the accumulation of total phenolics^66^, which aid in the scavenging of reactive oxygen species (ROS) that are known to cause cell damage under environmental stress^67^. In this study, T2 recorded the highest values for the total phenolic content (TPC) followed by T3 and T1, respectively, in both cuts. This means that plants in T2 were under stress that could have been caused by clogging of plant roots or limited water supply. Biofloc wastewater is known to contain high concentrations of particulate organic matter that clogs irrigation pipes and roots, thus affecting the growth performance of plants^68^. Clogging of plant roots interferes with root respiration as well as the uptake of water and mineral elements from the soil, consequently leading to plant stress^69^. Furthermore, wastewater from fish production systems such as Biofloc technology (BFT) and aquaculture is known to contain a high microbial load that is beneficial to the plants. These microbes have been reported to induce the production and accumulation of total phenolics in plant tissues^70^.

Besides total phenolics, plants also biosynthesize and accumulate essential oils (EOs) in their tissues as a response to several factors such as environmental stress and agronomical practices^71^. In this study, higher EO yields were obtained in cut 2 than in cut 1 across all treatments. Agronomical practices such as multiple cuttings not only induce stress but also enhance the vegetative growth of the plant, which is positively correlated with the EO yield^42,48,50^. Hence, more cuttings usually lead to higher EO yields, as has been reported by Barut et al.^72^, Zheljazkov et al.^73^, and Kimera et al.^50^. The major EO constituents across all treatments were 4-Terpineol (33.95% − 50.24%), γ-Terpinene (3.95% − 15.74%), α-Humulene (3.39% − 10.33%), α-Terpineol (3.72% − 8.37%), and Sabinene (2.16% − 6.31%). 4-Terpineol has been previously reported as the most abundant EO constituent in *O. majorana*^2,50,74^. However, the percentage composition reported in this study and the previous studies is quite above the acceptable limits (0.2% − 1.2%) as per the International Organization for Standardization (ISO 4728:2003)^2^. T1 recorded the highest total percentage of major EO constituents, followed by T2 and T3, respectively, thus highlighting the importance of plant nutrition in the production of MAPs. Plant nutrition influences the activity of several enzymes involved in the biosynthesis of certain compounds; however, other parameters such as the species, growth stage, origin of the herb, drying and climatic conditions contribute to the variation in EO yield and composition^75^. For instance, *O. majorana* harvested in Tunisia comprised Terpinen-4-ol (29.13–32.57%), cis-sabinene hydrate (19.9–29.27%) and trans-sabinene hydrate (3.5–11.61%)^3^ whereas in Spain, the major EO constituents of *O. majorana* were 1,8-cineole (58.59%), linalool (13.05%) and a-terpineol (3.33%).

## Conclusion and future research perspectives

Our results indicate that plant nutrition is a very important factor in the production of medicinal and aromatic plants. Chemical fertilization of *Origanum majorana* L. under greenhouse conditions enhances the vegetative growth of the plant, which also improves the essential oil yield and composition, especially 4-Terpineol, γ-Terpinene, α-Humulene, α-Terpineol, and Sabinene. In light of this investigation, it is also evident that, irrespective of the treatments, the second cut generally leads to higher essential oil yields than the first cut. Likewise, irrigating *O. majorana* with Biofloc wastewater enhances the total phenolic content in plant tissues. The results of this study provide relevant information for the cosmetics, pharmaceutical, and agricultural sectors regarding the use of water of different qualities in the greenhouse production of *O. majorana*. Conversely, more studies are needed to elucidate the influence of Biofloc and aquaculture wastewater on physiological responses, biochemical pathways and differential gene expression of *O. majorana* cultivated under our experimental conditions.

## Supplementary Information

Below is the link to the electronic supplementary material.

Supplementary Material 1

## Data Availability

The datasets used and/or analyzed during the current study are provided within the manuscript or supplementary information files.
