# Supplementary figures and images for: Comparative analysis of plant morphometric traits, essential oil yield, and quality of Origanum majorana L. cultivated under diverse sustainable organic nutrient management strategies
Source: Sci Rep. 2025 Sep 25;15:32934. doi: 10.1038/s41598-025-20751-x (PMC12464189; doi:10.1038/s41598-025-20751-x)

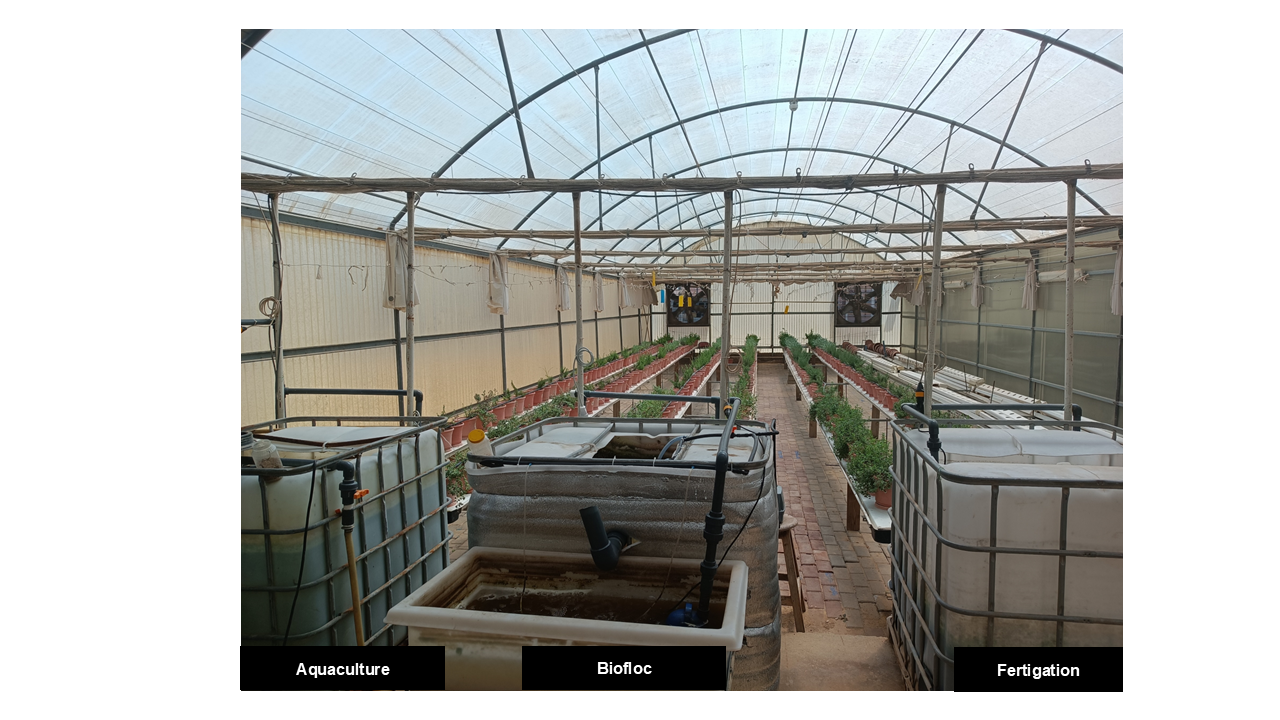


**Supplementary Fig. 1**. Greenhouse layout of the experimental treatments.

Supplement: Supplementary file 1 — Supplementary Material 1 [file 41598_2025_20751_MOESM1_ESM.docx]
